# *Aspergillus labruscus* sp. nov., a new species of *Aspergillus* section *Nigri* discovered in Brazil

**DOI:** 10.1038/s41598-017-06589-y

**Published:** 2017-07-24

**Authors:** Maria Helena P. Fungaro, Larissa S. Ferranti, Fernanda P. Massi, Josué J. da Silva, Daniele Sartori, Marta H. Taniwaki, Jens C. Frisvad, Beatriz T. Iamanaka

**Affiliations:** 1 Centro de Ciências Biológicas, Universidade Estadual de Londrina, Londrina, Paraná Brazil; 2 Centro de Ciência e Qualidade de Alimentos, Instituto de Tecnologia de Alimentos, Campinas, São Paulo Brazil; 3 Department of Biotechnology and Biomedicine, Technical University of Denmark, Lyngby, Denmark

## Abstract

A novel fungal species, *Aspergillus labruscus* sp. nov., has been found in Brazil during an investigation of the fungal species present on the surface of grape berries (*Vitis labrusca* L.) for use in the production of concentrated grape juice. It seems to be associated to *V*. *labrusca*, and has never been recovered from *Vitis vinifera*. This new species belonging to *Aspergillus* subgenus *Circumdati* section *Nigri* is described here using morphological characters, extrolite profiling, partial sequence data from the *BenA* and *CaM* genes, and internal transcribed spacer sequences of ribosomal DNA. Phenotypic and molecular data enabled this novel species to be clearly distinguished from other black aspergilli. *A*. *labruscus* sp. nov. is uniseriate, has yellow mycelium, poor sporulation on CYA at 25 °C, abundant salmon to pink sclerotia and rough conidia. Neoxaline and secalonic acid D were consistently produced by isolates in this taxon. The type strain of *A*. *labruscus* sp. nov. is CCT 7800 (T) = ITAL 22.223 (T) = IBT 33586 (T).

## Introduction

The production and commercialization of grape juice concentrate is growing every year in Brazil. Purple grape juice is rich in polyphenol compounds which have been associated with protective effects on the vascular system and with improvement in cognition and neuronal function with aging^1^.

Serra Gaúcha, in the southern Brazilian state of Rio Grande do Sul is acknowledged as the largest grape producing region in Brazil. The main features of this region include low levels of sunlight and acid soils with good drainage. Various grape cultivars are widely used in the Serra Gaúcha region, but some 90% of the growing area is planted with vines of *Vitis labrusca* L., the most important species for grape juice production^2^. Grapes have been grown in this region since the 19^th^ century when Italian immigrants arrived bringing with them the culture and tradition of wine production and consumption. These Italian immigrants used their farming knowledge in their new homeland to provide food for consumption and subsistence. However, the climatic conditions of the region were not favorable for growing the European (*Vitis vinifera* L.) grape cultivars, which led to the introduction of hardier cultivars of American origin (*V. labrusca*) with better resistance to biotic and abiotic stress. Nowadays “Isabel”, “Isabel Precoce”, “Bordô” and “Concord” are the *V*. *labrusca* cultivars extensively grown throughout Rio Grande do Sul. In 2014, 540,000 Kg of *V*. *labrusca* grapes were produced in this state^3^, and used mostly for the production of concentrated grape juice.

More recently, in addition to Rio Grande do Sul, the Brazilian states of Pernambuco (Petrolina region), Paraná, São Paulo and Minas Gerais have also become significant producers of grape concentrate for juice. This kind of juice is exported to many countries, and used to make grape nectar and grape beverages. Indeed, Brazil is the 10^th^ largest exporter by volume of grape juice and is one of the few countries in the world where *V*. *labrusca* is grown on a commercial scale. About 90 million liters of grape juice were sold in 2014.

Fungi belonging to *Aspergillus* section *Nigri*, also called “the black aspergilli”, have been shown to occur frequently throughout the world on grapes for wine production, such as *V*. *vinifera* ^4–8^. However, black aspergilli populations on grapes for the production of concentrated grape juice, and especially *V*. *labrusca*, have yet to be described. Some black aspergilla are important in biotech processes and also in biodeterioration, but some species in this section can produce carcinogenic mycotoxins ochratoxin A^9^ and fumonisins, especially fumonisin B_2_^10^.

Samson *et al*.^11^ recently provided an updated accepted species list for the genus *Aspergillus*, and 27 species are included in *Aspergillus* section *Nigri*. The authors discussed different approaches to species identification in *Aspergillus* and recommended DNA markers as a reliable means of identification. Information on living ex-type culture collection numbers and GenBank accession numbers for available representative ITS, *CaM*, *BenA* and *RPB2* sequences were listed, and *CaM* sequences were proposed as important identification markers for *Aspergillus* section *Nigri*.

During an investigation of the fungal species in grapes for juice production, a new *Aspergillus* taxon in *Aspergillus* section *Nigri* was found on the surface of grape berries. This species was found only in the region of Serra Gaúcha, Rio Grande do Sul, and on one grape variety, “Bordô”. It is described here as *Aspergillus labruscus* sp. nov. and this species does not produce ochratoxins or fumonisins.

## Results and Discussion

### Molecular identification

It is very difficult to identify fungi belonging to *Aspergillus* section *Nigri* due to the subtle morphological differences between species, and therefore DNA sequence information is increasingly being used for species identification and diagnosis.

Fungi belonging to section *Nigri* have been identified mainly using *CaM* gene sequences, because the internal transcribed spacer (ITS) of the nuclear ribosomal (nrDNA), accepted as the official DNA barcode for fungi^12^, is insufficient for correctly identifying all *Aspergillus* section *Nigri* species^11^. The *CaM* gene sequence contains more variation than the ITS and the nucleotide sequence database is complete for all accepted species^11^.

Thus, as an initial step, part of the *CaM* gene sequence was determined for 275 isolates of *Aspergillus* section *Nigri* found on the surface of the *V*. *labrusca* grape berries for four different Brazilian regions, to identify the species. All *CaM* gene sequences were compared using the Basic Local Alignment Search Tool (BLAST) against the NCBI database to recognize fungal species that have similar DNA sequences. The majority of gene sequences were similar (sequence identity ≥99%) to one of the 27 species belonging to *Aspergillus* section *Nigri*, but the *CaM* gene sequences from 23 isolates were found to be significantly different from all other sequences of species described so far (NCBI accessed 20 Dez, 2016). Using the BLAST tool it was found that the *CaM* gene sequences from these isolates are most similar to those from *Aspergillus homomorphus*, but with only 85% of sequence identity.

**Figure 1:**
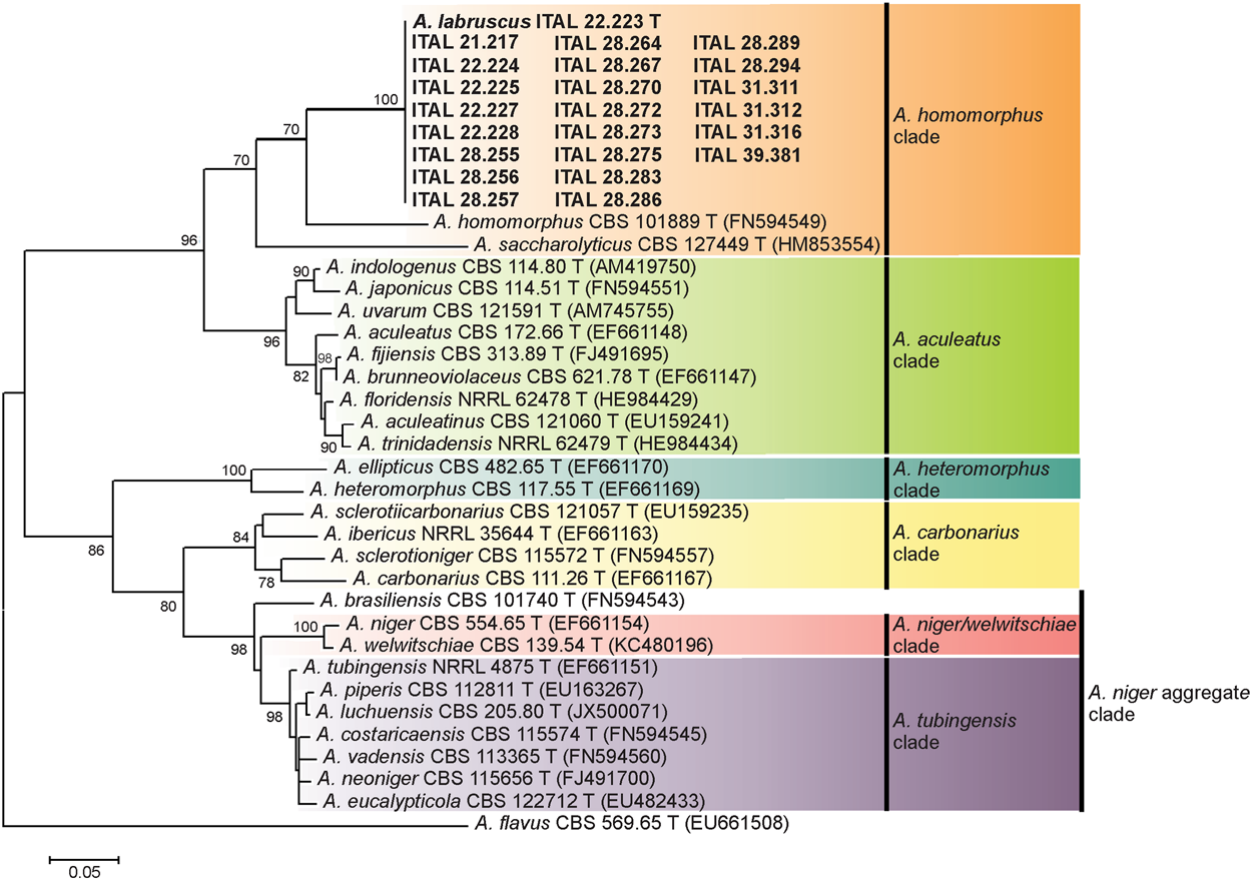
Maximum Likelihood phylogenetic tree based on partial calmodulin gene (*CaM*) sequence data for *Aspergillus* section *Nigri*. Bootstrap percentages (based on 1,000 resampled datasets) are shown at nodes. *Aspergillus flavus* was used as outgroup.

Samson *et al*.^11^ provided an updated accepted species list for the genus *Aspergillus*, now containing 339 species, and to enhance the scientific value of the list, it includes information on living ex-type culture collection numbers and GenBank accession numbers for available representative ITS, *CaM*, *BenA* and *RPB2* sequences. We generated a *CaM*-based phylogram of the 23 sequences not assigned to any described species using the BLAST tool, and those retrieved from GenBank for each *Aspergillus* section *Nigri* on Samson’s updated list. The *CaM*-based phylogram placed our isolates on a branch clearly separated from all other species of *Aspergillus* section *Nigri* (Fig. 1). The novel isolates were found to belong to a clade including *A*. *homomorphus* and *Aspergillus saccharolyticus*.

**Figure 2:**
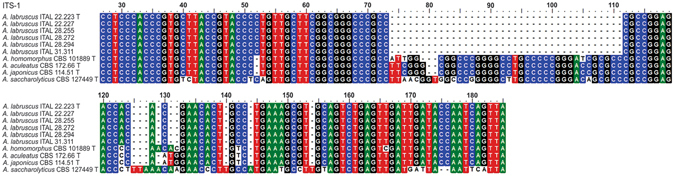
Sequence alignment of ITS1 from *Aspergillus labruscus*, *A. aculeatus, A. homomorphus, A. japonicus* and *A. saccharolyticus*.

Six isolates of the putative novel species were randomly selected for further analysis. The nrDNA (ITS), region and partial *BenA* gene were sequenced. As discussed by Samson *et al*.^11^, a frequent difficulty experienced with sequence-based identifications is comparing newly obtained sequences with verified sequence databases. GenBank is a public, archival database, which means that it accepts all sequences submitted and cannot always verify the taxonomic names attributed to the sequences, and therefore BLAST search results may give hits to misidentified sequences in the database. In an attempt to clean up misidentified GenBank sequences, the RefSeq initiative was launched (http://www.ncbi.nlm.nih.gov/refseq/), and lists only verified sequences^13^. For *Aspergillus*, all ex-type sequences were included in the RefSeq database. Using the BLAST tool with the “sequences from type material” (RefSeq) option (accessed 20 Jan, 2017), the ITS sequence from our novel isolates was found to be most similar to those from *A*. *homomorphus* CBS 101889, *Aspergillus aculeatus* CBS 172.66 and *Aspergillus japonicus* CBS 114.51, all of which have only 85–88% sequence identity. Both, *A*. *aculeatus* and *A*. *japonicus* belong to the *A*. *aculeatus* clade in *Aspergillus* section *Nigri*, and *A*. *homomorphus* belongs to the *A*. *homomorphus* clade in section *Nigri* ^11^. Interestingly, the ITS amplicon length from all isolates of the putative novel species (540 bp), obtained with the ITS1-ITS4 primer-pair^14^, was smaller than that of all other *Aspergillus* section *Nigri*. They exhibit a 38–39 bp deletion in the ITS1 region when compared to *A*. *aculeatus*, *A*. *homomorphus*, *A*. *japonicus* and *A*. *saccharolyticus* (Fig. 2).

Although the *BenA* gene may vary in the number of introns, and the PCR sometimes results in the amplification of paralogous genes^15^, this *locus* has been frequently used for molecular identification of *Aspergillus*. Thus, the DNA sequences from the six isolates of the putative novel species were determined for a portion of *BenA*. Comparative BLAST sequence analyses of the six isolates revealed that they are more similar to *A*. *homomorphus* ex-type strain CBS 101889, with 85% of sequence identity. A *BenA*-based phylogram placed the six isolates on a branch undoubtedly separated from all other species of *Aspergillus* section *Nigri* (Supplementary Figure 1).

In conclusion, the ITS, *BenA* and *CaM* sequences harmonized to show that our isolates belong to a novel phylogenetic species. We suggest the name *Aspergillus* *labruscus* sp. nov. for this new phylogenetic species and as its type, strain CCT 7800 (T) = ITAL 22.223 (T) = IBT 33586 (T). The GenBank accession numbers of the ITS barcodes and alternative identification markers *Ben* *A* and *CaM* deposited for this paper are present in Table 1.

**Table 1:** GenBank accession numbers of the sequences deposited for this paper.

Strain	Origen*	ITS	*BenA*	*CaM*
ITAL 22.223 (T) = IBT 33586 (T) = CCT 7800 (T)	Bento Gonçalves	KU708544	KT986014	KT986008
ITAL 22.227 = IBT 33583	Bento Gonçalves	KU708545	KT986015	KT986009
ITAL 28.255 = IBT 33584	Garibaldi	KU708546	KT986016	KT986010
ITAL 28.272 = IBT 33581	Garibaldi	KU708547	KT986017	KT986011
ITAL 28.294 = IBT 33585	Garibaldi	KU708548	KT986018	KT986012
ITAL 31.311 = IBT 33582	Veranópolis	KU708549	KT986019	KT986013

*Locality in Rio Grande do Sul, Brazil (City). T, type culture.

### Metabolite Analysis

Six strains of *A*. *labruscus* were examined for extrolites and they all produced secalonic acid D and neoxaline (Table 2). The phylogenetically closely related species *A*. *saccharolyticus* differed from *A*. *labruscus* by producing aculene A and B^16^ and homomorphosins. *A*. *homomorphus* ^17^ shares production of secalonic acid D with *A*. *labruscus*, but differs by producing homomorphosins and 3-methoxy-5-hydroxy-9-phenyl-2,4,6,8-nanotetranoic acid lactone (Hoeck, Petersen, Frisvad, Gotfredsen and Larsen, personal communication). The ex type strain produced two as yet not structure elucidated extrolites, that have only been observed in *A*. *labruscus*. Metabolite analysis indicated that *A*. *labruscus* does not produce ochratoxins and fumonisins.

**Table 2:** Extrolite profiles of *Aspergillus labruscus* strains*.

Strain	Extrolite profile
ITAL 22.223 (T) = IBT 33586 (T) = CCT 7800 (T)	neoxaline, secalonicacid D
ITAL 28.294 = IBT 33585	neoxaline, secalonic acid D
ITAL 22.227 = IBT 33583	neoxaline, secalonic acid D
ITAL 31.311 = IBT 33582	neoxaline, secalonic acid D an apolar aculene (with an aculene A chromophore)
ITAL 28.272 = IBT 33581	neoxaline, secalonic acid D
ITAL 28.255 = IBT = 33584	neoxaline, secalonic acid D

*Strain ITAL 28.275, ITAL 22.225 and ITAL 28.256 also produced secalonic acid D and neoxaline.

### Macro and Micromorphology Analysis

**Figure 3:**
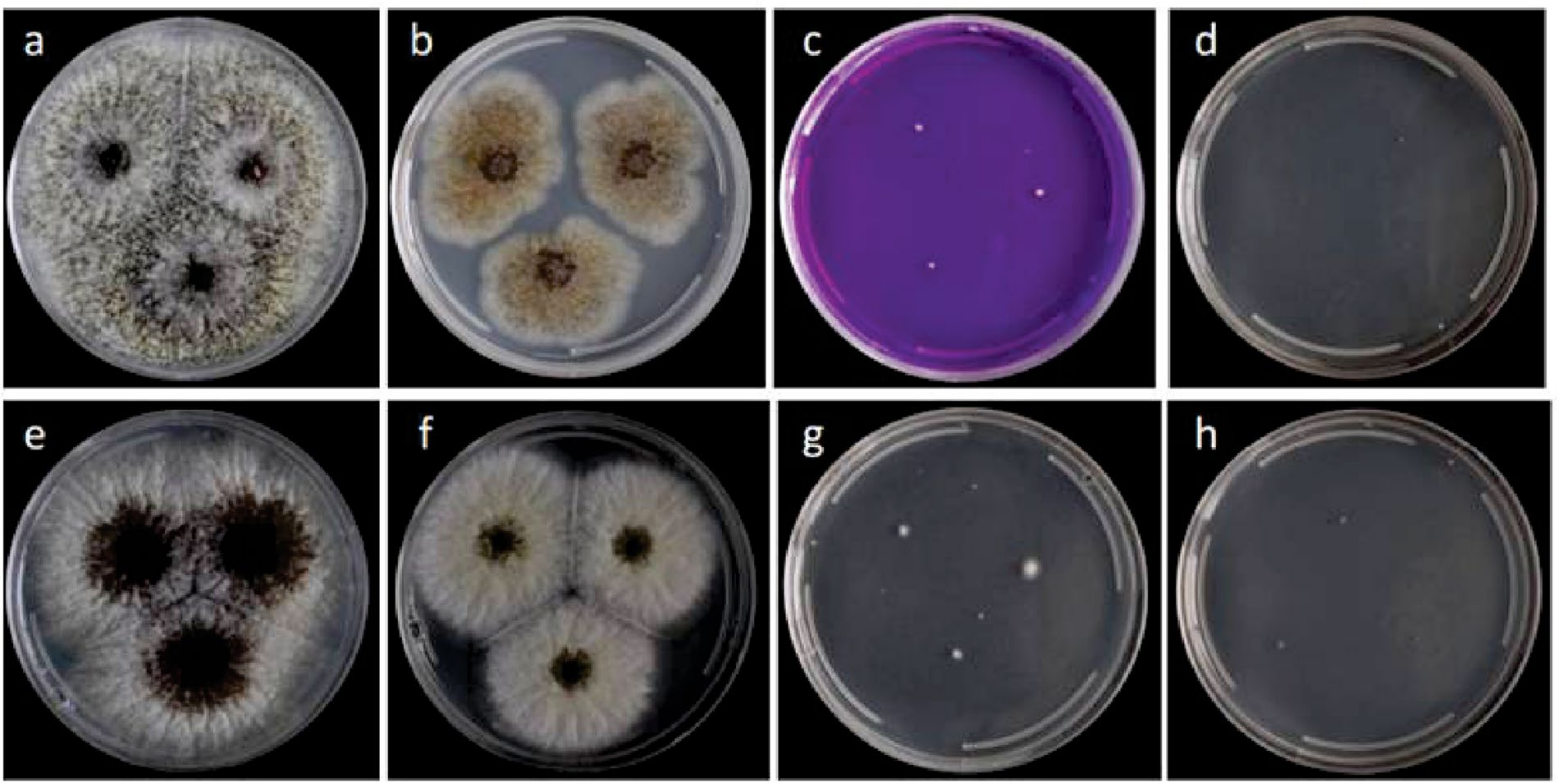
*Aspergillus labruscus* sp. nov. CCT 7800. (**a**) Colonies on CYA at 25 °C, (**b**) MEA at 25 °C, (**c**) CREA at 25 °C, (**d**) CYA at 33 °C, (**e**) CYA at 30 °C, (**f**) CYA at 20 °C, (**g**) CYA at 15 °C and (**h**) CYA at 10 °C.

**Figure 4:**
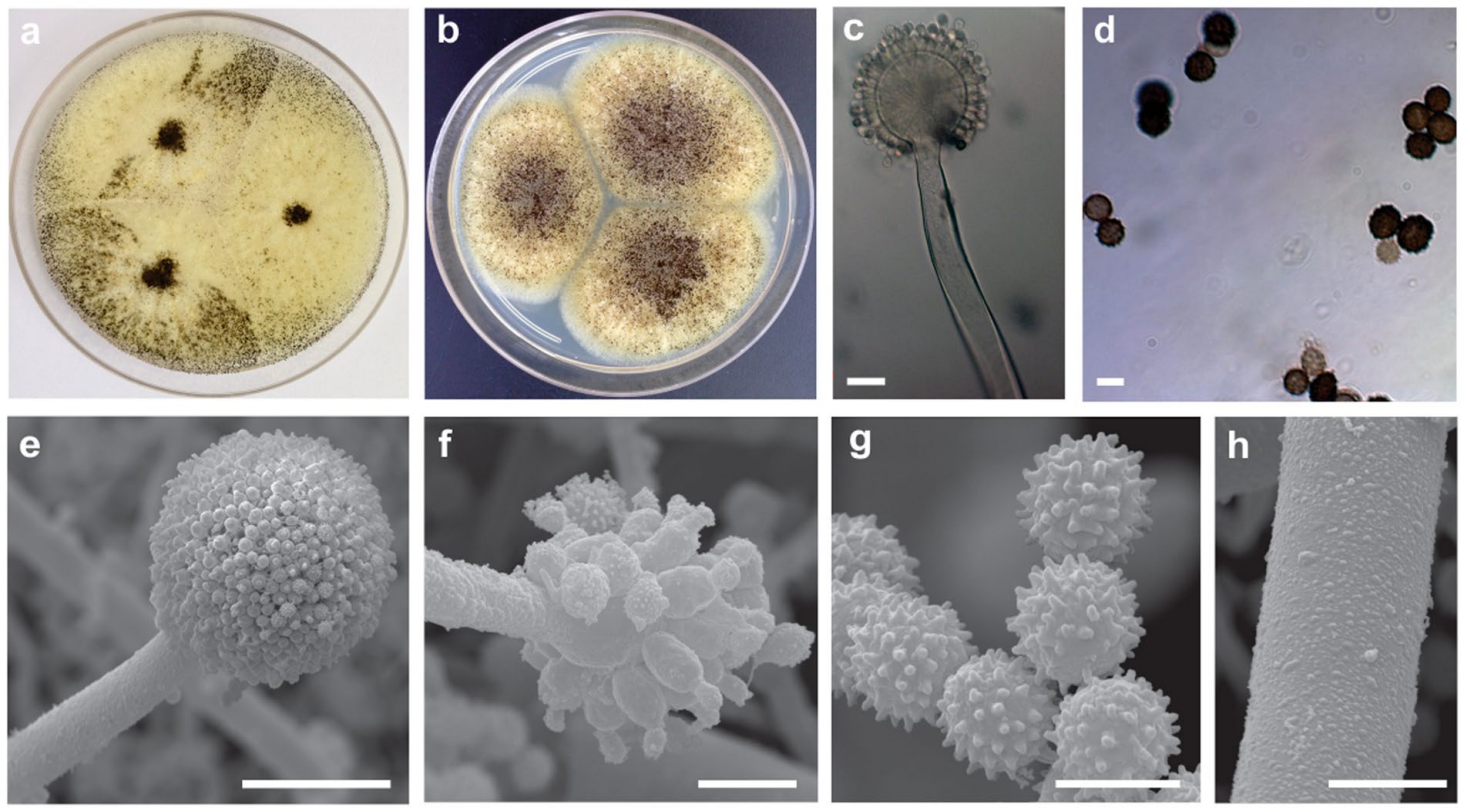
*Aspergillus labruscus* sp. nov. CCT 7800. (**a**) Colonies on CYA, (**b**) colonies on MEA, (**c**) conidiophores under light microscopy, (**d**) conidia under light microscopy, (**e**,**f**) conidiophores as seen using SEM, (**g**) conidia as seen using SEM, (**h**) stipe as seen using SEM. Bars, 10 µm (**c**,**f** and **h**), 5 µm (**d** and **g**), 50 µm (**e**).

Colony diameters after 7 days at 25 °C were as follows: CYA: 70–77 mm, MEA: 56–60 mm, YESA: 69–79 mm, OAT: 42–45 mm, CREA: 3–4 mm and CYA + 5% NaCl: no growth. On CYA at 10 °C conidia germinate, at 15 °C, 20 °C and 30 °C growth is evident with colony diameters of 9–17 mm, 59–71 mm and 56–86 mm, respectively. The species does not grow on CYA at 37 °C and 42 °C while at 33 °C the colony diameters vary from 0 to 22 mm, indicating that at this temperature the growth response varies among the isolates. At 25 °C in CYA, the species showed yellow mycelium, poor sporulation, abundant salmon to pink sclerotia production, and reverse pale yellow in color. Figure 3 shows the colonies of *A*. *labruscus* (CCT 7800) on different media and temperatures. Micromorphology characters are as follows: uniseriate, spherical conidial heads, brown colored stipes smooth with thick wall (3.2–6.1 µm), vesicle 43 × 60 µm, phialides 7.2–7.8 × 3.8 µm, conidia not uniform in size and shape, spherical to ellipsoidal 6.5–8 × 6.1–6.9 µm, black and conspicuously rough. Figure 4 shows the morphology on CYA and MEA at 25 °C and the micromorphological characteristics.

Morphologically *A*. *labruscus* is similar to *Aspergillus sclerotioniger* because both have yellow mycelium, poor sporulation on CYA at 25 °C, abundant sclerotia and rough conidia. However, they differ because *A*. *sclerotioniger* has biseriate conidiophore, yellow sclerotia and produces ochratoxin A^11^ while *A*. *labruscus* is uniseriate, has pink to salmon sclerotia and does not produce this mycotoxin.

Although molecular data revealed that *A*. *labruscus* is most similar to *A*. *homomorphus*, *A*. *saccharolyticus* and *A*. *aculeatus*, the yellow mycelium, salmon to pink sclerotia and large spherical to ellipsoidal conidia (6.5–8 µm) differentiate *A*. *labruscus* from these species. Furthermore, *A*. *labruscus* differed from *A*. *homomorphus* and *A*. *saccharolyticus* because these two species were able to grow on CYA at 37 °C with diameters 18 mm and 7–10 mm, and on CYA + 5% NaCl with diameters of 68 mm and 17 mm, respectively.

### Incidence and Ecology

Among a total of 89 grape samples collected from different regions and grape cultivars analyzed for fungal contamination, 5 samples (5.6%) revealed the presence of *A*. *labruscus*. In total, 23 isolates of this new species were found on the surface of grape berries, *V*. *labrusca*, cv. Bordô grown in Rio Grande do Sul (Serra Gaúcha). The origins of representative *A*. *labruscus* isolates are shown in Table 3. This new species was not found in the other Brazilian regions. Table 4 shows the frequency of *A*. *labruscus* on the samples from different vineyards of Rio Grande do Sul and the range of contamination. Serra Gaúcha has a distinct climatic condition for vineyards, with an annual average temperature of 17 °C, rainfall of 1,700 mm and relative humidity of 76%. While most of the world’s vineyards grow the European cultivars (*V*. *vinifera*) which produces fine wine, in Brazil due to the high humidity conditions, this species was affected by fungal disease and did not adapt to Brazilian conditions. Thus, the rustic North American cultivars of *V*. *labrusca* were introduced, which showed high disease resistance, and today 80% of Brazilian vineyards grow the cultivar *V*. *labrusca*, especially in the State of Rio Grande do Sul.

**Table 3:** *Aspergillus labruscus* strains from grapes grown in Rio Grande do Sul State.

*ITAL Code	**CCT	***IBT	Cultivar	Region (City/State)
21.217			Bordô	Bento Gonçalves/RS
22.223	7800	33586	Bordô	Bento Gonçalves/RS
22.224			Bordô	Bento Gonçalves/RS
22.225		33589	Bordô	Bento Gonçalves/RS
22.227		33583	Bordô	Bento Gonçalves/RS
22.228			Bordô	Bento Gonçalves/RS
28.255		33584	Bordô	Garibaldi/RS
28.256		33587	Bordô	Garibaldi/RS
28.257			Bordô	Garibaldi/RS
28.264			Bordô	Garibaldi/RS
28.267			Bordô	Garibaldi/RS
28.270			Bordô	Garibaldi/RS
28.272		33581	Bordô	Garibaldi/RS
28.273			Bordô	Garibaldi/RS
28.275		33588	Bordô	Garibaldi/RS
28.283			Bordô	Garibaldi/RS
28.286			Bordô	Garibaldi/RS
28.289			Bordô	Garibaldi/RS
28.294		33585	Bordô	Garibaldi/RS
31.311			Bordô	Veranópolis/RS
31.312			Bordô	Veranópolis/RS
31.316			Bordô	Veranópolis/RS
39.381			Bordô	Pinto Bandeira/RS

*ITAL = culture collection of the Instituto de Tecnologia de Alimentos, Campinas, Brazil. **CCT = tropical culture collection of André Tosello Foundation, Campinas, Brazil. ***IBT = culture collection of the Technical University of Denmark, Lyngby, Denmark.

**Table 4:** Incidence of *Aspergillus labruscus* on grape berry samples (*Vitis labrusca*) from Rio Grande do Sul, Brazil.

Origin	N° of positive samples/n° of samples	Range of contamination (%)
Bento Gonçalves	2/9	0–5%
Garibaldi	1/3	0–13%
Veranópolis	1/6	0–3%
Pinto Bandeira	1/5	0–1%
Farroupilha	0/4	0
Caxias do Sul	0/3	0
Sample Total	5/30	0–15%

It is interesting to note that *A*. *labruscus* has never been found on *V*. *vinifera* grapes. A similar situation is known for peanuts where the species *Aspergillus arachidicola*, has only been found on the wild type species *Arachis glabrata*, but not on the domesticated peanut, *Arachis hypogea* ^18^.

Data on growth temperatures under laboratory condition as presented above showed that *A*. *labruscus* was able to grow at temperatures of 15 °C to 33 °C, with no growth at 37 °C. Most members of *Aspergillus* section *Nigri* can grow at 37 °C and even higher^19, 20^, including the closely species *A*. *homomorphus* and *A*. *saccharolyticus*. *A*. *labruscus* has never been found before in European vineyards and grape products^21^ indicating that *A*. *labruscus* have an association with *V*. *labrusca* rather than *V*.*vinifera*. Data on the growth temperature of *A*. *labruscus* show that this species is adapted to colder temperatures, which may be due to its North American origin and therefore well-adapted to the Serra Gaúcha region which is colder than the other regions in Brazil. In addition, data to be published later will show that this species has not been found in *V*. *labrusca* cv. Bordô vineyards in the North Brazilian region.

### Enzyme production

Black aspergilli are particularly effective enzyme producers^20^ and the three closely related species *A*. *labruscus*, *A*. *homomorphus* and *A*. *saccharolyticus* were examined for the production of two extracellular enzymes: tannase and caseinase (Table 5). On TAN medium used for tannase production, *A*. *labruscus*, *A*. *homomorphus* and *A*. *saccharolyticus* grew and showed good sporulation, but the tannase production was most pronounced in *A*. *homomorphus*, followed by *A*. *labruscus* and was poor in *A*. *saccharolyticus*. Wine fruits have a high content of tannin, and it would be expected that *A*. *labruscus* can produce tannase. On Pro agar, the three species grew and sporulated well, but caseinase was not produced by *A*. *homomorphus* while *A*. *labruscus* and *A*. *saccharolyticus* showed very good production of this enzyme. These two culture media for extracellular enzyme production showed good performance to differentiate these three species, showing that they are distinct and giving a different response in enzyme production. Since *A*. *saccharolyticus* is one of the best β-glucosidase producers known^22, 23^, it should be investigated whether *A*. *labruscus* is also an effective producer of such enzymes.

**Table 5:** Growth, sporulation and production of extracellular enzymes of *Aspergillus labruscus*, *A*. *homomorphus* and *A*. *saccharolyticus*.

Culture media	Growth diameter (mm)		
	*A*. *labruscus*	*A*. *homomorphus*	*A*. *saccharolyticus*
**TAN**	18–32	38	24–38
Sporulation	++	++	++
Tannase	+	+	±
**Pro agar**	80	80	57–67
Sporulation	+++	+++	++
Caseinase	+++	−	+++
**CYA with raisins**	80	80	80
Sporulation	+++	++	++
Sclerotia	+++	−	±

−None, ±Poor, +Some, ++Good, +++Very good.

### Taxonomy

*Aspergillus labruscus* Fungaro, Sartori, Ferranti, Frisvad, Taniwaki, Iamanaka sp. nov. (Fig. 4).

MYCOBANK: MB815746

Etymology**:** “labrusca” is Latin for wild grape vine, on which this fungus species was found.

- **Holotype:** CCT 7800, a freeze dried culture in Tropical Culture Collection of André Tosello Foundation (Campinas, Brazil), is designated as the holotype of *A*. *labruscus*. It was isolated from the surface of grape berries (*Vitis labrusca*) grown in Rio Grande do Sul State, Brazil, in February, 2013, by Fungaro MHP and Sartori, D. Cultures derived from this type include ITAL 22.223 (ITAL = culture collection of the Instituto de Tecnologia de Alimentos, Campinas, Brazil) and IBT 33586 (IBT = culture collection of the Technical University of Denmark, Lyngby, Denmark).
- **Diagnosis:** This species differs from all species in *Aspergillus* section *Nigri* ^24^ by its yellow mycelium, poor sporulation on CYA at 25 °C, abundant salmon to pink sclerotia and no growth at 37 °C on CYA. A profile of secondary metabolites differing from that of the closely related species *A*. *homomorphus* and *A*. *saccharolyticus* and a distinctive DNA sequence of *BenA* and *CaM* genes and ITS regions.
- **Description:** Colony on CYA, after 7 days at 25 °C: 73–84 mm diameter, yellow mycelium, poor sporulation, abundant salmon to pink sclerotia, and pale yellow reverse. On MEA, after 7 days at 25 °C: colony of 58–78 mm diameter, abundant sporulation and pale yellow reverse. The species does not grow on CYA at 37 °C and CYA at 42 °C after 7 days, but can grow on CYA at 15 °C (diameter of 9–17 mm) and germinate at 10 °C. Conidiophores uniseriate with spherical vesicles 40 × 60 µm, stipe smooth-walled and brown colored 3.2–6.1 µm, phialides 7.2–7.8 × 3.8 µm, conidia not uniform in size and shape, spherical to ellipsoidal with 6.5–8.0 µm, black and coarsely roughened.
- **ITS Barcode:** KU708544 (alternative markers: *BenA* = KT986014; *CaM* = KT986008).
- **Extrolites:** All isolates produced secalonic acid D and neoxaline.
- **Other isolates examined:** ITAL 21.217, ITAL 22.224, ITAL 22.225, ITAL 22.227, ITAL 22.228, ITAL 28.255, ITAL 28.256, ITAL 28.257, ITAL 28.264, ITAL 28.267, ITAL 28.270, ITAL 28.272, ITAL 28.273, ITAL 28.275, ITAL 28.283, ITAL 28.286, ITAL 28.289, ITAL 28.294, ITAL 31.311, ITAL 31.312, ITAL 31.316, ITAL 39.381 all from the surface of grape berries (*V*. *labrusca*) grown in the Rio Grande do Sul State, Brazil.

## Methods

### Grape samples and mycological analysis

The 89 grape samples were collected from four Brazilian states: Pernambuco (*n* = 22), São Paulo (*n* = 21), Paraná (*n* = 16) and Rio Grande do Sul (*n* = 30). In 2014, twenty bunches were collected from each field, close to harvest time between late January and early February in Paraná and Rio Grande do Sul, in April in Pernambuco, and in December in São Paulo. The *V*. *labrusca* grape cultivars studied were “Bordô”, “Cora”, “Concord”, “Isabel”, “Isabel Precoce”, “Violeta”, “Coder”, “Rudder”, “Niagara” and “Muscadine”. These are the grape cultivars most widely used for juice production in Brazil. The samples were collected as described in Serra *et al*.^25^, across two diagonal transects. A total of 100 berries of each sample was plated according to Pitt and Hocking^26^ but without surface disinfection. All *Aspergillus* section *Nigri* fungi were transferred to CYA (Czapek Yeast Extract agar, formulated) and incubated at 25 °C for seven days.

### Molecular analysis

A total of 275 *Aspergillus* section *Nigri* isolates obtained from the grapes were randomly selected and subjected to DNA extraction using a commercial extraction kit, Biopur Mini Spin Planta (Biopur, Brazil), according to the manufacturer’s instructions. As an initial step to identify the isolates, partial amplification and sequencing of the *CaM* gene were performed as described in detail by Taniwaki *et al*.^27^. The *CaM* sequences obtained were aligned with type or neotype strain sequences from all *Aspergillus* section *Nigri* species obtained from the database maintained by NCBI (http://www.ncbi.nlm.nih.gov/). Alignment was performed using ClustalW^28^. MEGA6.0 software^29^ was used to construct a Maximum Likelihood (ML) tree, based the Tamura-Nei model^30^. Of the twenty three isolates none were phylogenetically closely related to any of the species described so far, six were further investigated using the ITS1–5.8S–ITS2 region of nrDNA (ITS) and partial *BenA* gene sequences. Partial amplification and sequencing of the *BenA* gene and ITS region were performed as described in Taniwaki *et al*.^27^. The ITS and *BenA* sequences obtained were aligned with the type or neotype strain sequences from all *Aspergillus* section *Nigri* species using the above-mentioned approaches.

### Extrolite analysis

Six isolates of *A*. *labruscus* (Table 2), two isolates of *A*. *homomorphus* (IBT 21893 and IBT 21894) and two isolates of *A*. *saccharolyticus* (IBT 28231 and IBT 30881 = CBS 127449) were analysed for small molecule extrolites by extracting 3 agar plugs from 7 day incubated cultures at an incubation temperature of 25 °C in darkness^31^. The extracts were analyzed by ultra high performance liquid chromatography using diode array detection (UHPLC-DAD)^32^ and compared to authentic small molecule extrolite standards^33^.

### Morphological characterization

Morphological characterization was performed based on Samson *et al*.^11^. Briefly, a spore suspension was prepared in 0.5% agar dissolved in water. Petri dishes were inoculated in a three-point pattern. The following culture media (formulated) and incubation temperature were used: Czapek yeast autolysate (CYA) agar at 10 °C, 15 °C, 20 °C, 25 °C, 30 °C, 33 °C, 37 °C, and 42 °C; malt extract agar (MEA), oatmeal agar (OAT), creatine sucrose agar (CREA) and CYAS (Czapek yeast autolysate agar with 5% NaCl), at 25 °C. For comparison, two isolates of *A*. *homomorphus* (IBT 21893 and IBT 21894) and two isolates of *A*. *saccharolyticus* (IBT 28231 and IBT 30881 = CBS 127449) were also grown on CYA at 37 °C and CYAS at 25 °C.

After seven days, macro and micromorphological characters of *A*. *labruscus* were examined under light optical microscopy and scanning electron microscopy. The slide for light optical microscopic observations was prepared using lactic acid and lactophenol cotton blue. For each structure 3 replicate measurements were performed. For scanning electron microscopy, a 0.5 × 0.5 cm plug was fixed for 24 h at 4 °C in 2% glutaraldehyde in 0.1 M of NaPO_4_ buffer. The plug was washed three times for 15 min (each) in 0.1 M phosphate buffer (NaPO_4_) and post-fixed in osmium tetroxide 1% buffer for 2 h at 25 °C in the dark. The plug was then washed again three times in 0.1 M phosphate (NaPO_4_) buffer for 15 min and dehydrated in ethanol series (70, 80, 90 and 100%) for 10 min. It was then placed in a critical point dryer (Bal-Tec, CSDC 030) and subsequently sputter-coated with gold (Bal-Tec, SDC 050). The observation was performed using a FEI Quanta 200® microscope.

### Enzyme production

Six isolates of *A*. *labruscus* (Table 2), two isolates of *A*. *homomorphus* (IBT 21893 and IBT 21894) and two isolates of *A*. *saccharolyticus* (IBT 28231 and IBT 30881 = CBS 127449) were tested for production of two extracellular enzymes: (i) tannase, growing the isolates on tannin sucrose agar (TAN) as described by Thrane^34^ and (ii) caseinase growing on PRO agar (skimmed milk powder 15%, 100 mL; glucose, 5.0 g; K_2_HPO_4_·3H_2_O, 1.0 g; KCl, 0.5 g; MgSO_4_·7H_2_O, 0.2 g; CaCl_2_·2H_2_O, 0.1 g; SM (trace metal solution), 1 mL; yeast extract, 3.0 g; agar, 20.0 g, water, 1 L). These isolates were one point inoculated on each medium and incubated at 25 °C for 7 days and examined for production of enzyme haloes.

## Electronic supplementary material

Supplementary Figure S1
