# Supplementary figures and images for: Aspergillus labruscus sp. nov., a new species of Aspergillus section Nigri discovered in Brazil
Source: Sci Rep. 2017 Jul 24;7:6203. doi: 10.1038/s41598-017-06589-y (PMC5524721; doi:10.1038/s41598-017-06589-y)

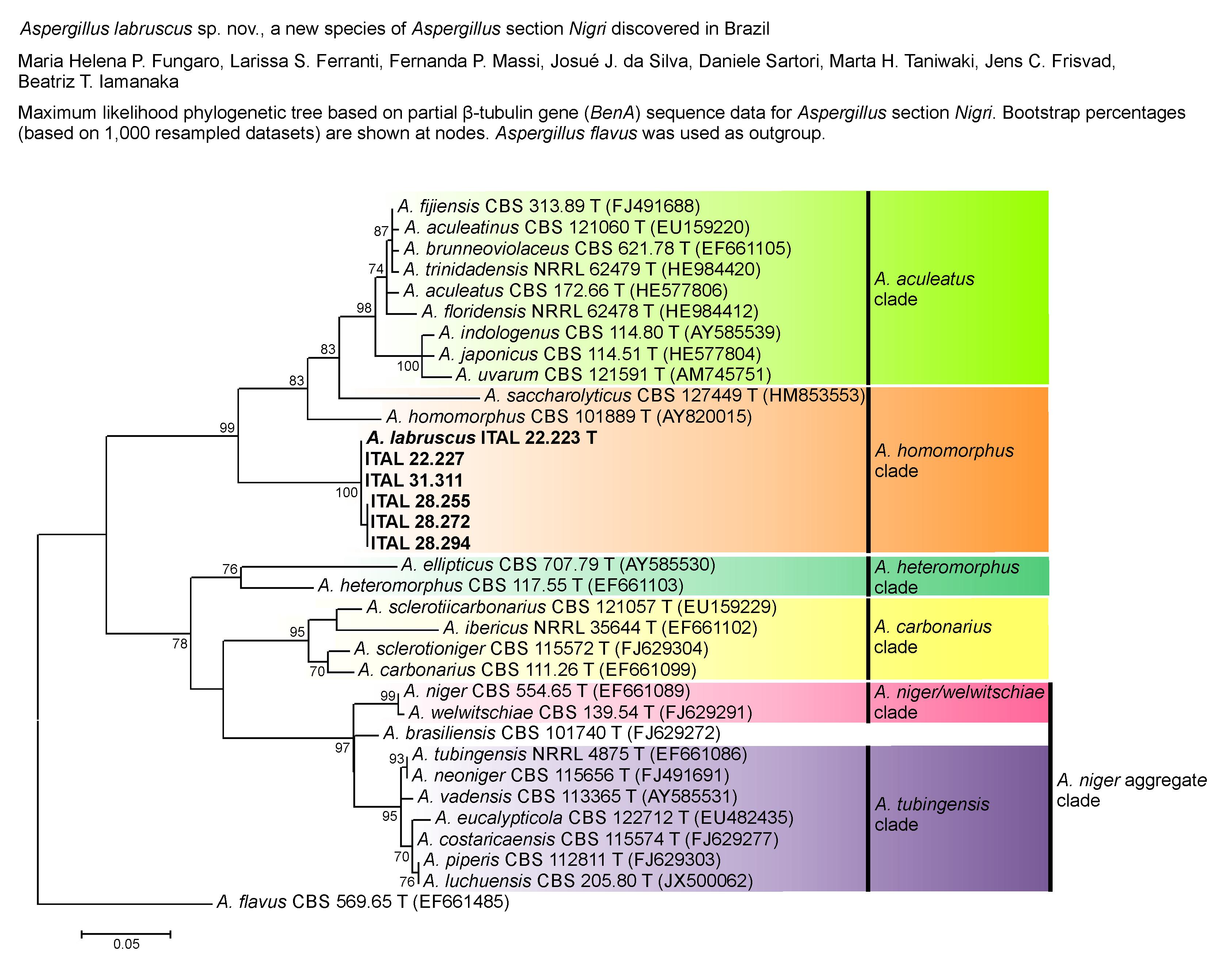

Supplement: Supplementary file 1 — Supplementary Figure S1 [file 41598_2017_6589_MOESM1_ESM.tif]
